# Chronic doxorubicin administration impacts satellite cell and capillary abundance in a muscle‐specific manner

**DOI:** 10.14814/phy2.14052

**Published:** 2019-04-08

**Authors:** Andrew C. D'Lugos, Christopher S. Fry, Jordan C. Ormsby, Kaylin R. Sweeney, Camille R. Brightwell, Taben M. Hale, Rayna J. Gonzales, Siddhartha S. Angadi, Chad C. Carroll, Jared M. Dickinson

**Affiliations:** ^1^ College of Health Solutions Arizona State University Phoenix Arizona; ^2^ Department of Nutrition and Metabolism University of Texas Medical Branch Galveston Texas; ^3^ Department of Basic Medical Sciences College of Medicine‐Phoenix University of Arizona Phoenix Arizona; ^4^ Department of Physiology Midwestern University Glendale Arizona; ^5^ Department of Health and Kinesiology Purdue University West Lafayette Indiana

**Keywords:** Chemotherapy, extensor digitorum longus, Ki67, Pax7, soleus

## Abstract

Anthracycline chemotherapies are effective at reducing disease recurrence and mortality in cancer patients. However, these drugs also contribute to skeletal muscle wasting and dysfunction. The purpose of this study was to assess the impact of chronic doxorubicin (DOX) administration on satellite cell and capillary densities in different skeletal muscles. We hypothesized that DOX would reduce satellite cell and capillary densities of the soleus (SOL) and extensor digitorum longus (EDL) muscles, along with muscle fiber size. Ovariectomized female Sprague‐Dawley rats were randomized to receive three bi‐weekly intraperitoneal injections of DOX (4 mg∙kg^−1^; cumulative dose 12 mg∙kg^−1^) or vehicle (VEH; saline). Animals were euthanized 5d following the last injection and the SOL and EDL were dissected and prepared for immunohistochemical and RT‐qPCR analyses. Relative to VEH, CSA of the SOL and EDL fibers were 26% and 33% smaller, respectively, in DOX (*P *<* *0.05). In the SOL, satellite cell and capillary densities were 39% and 35% lower, respectively, in DOX (*P *<* *0.05), whereas in the EDL satellite cell and capillary densities were unaffected by DOX administration (*P *>* *0.05). Proliferating satellite cells were unaffected by DOX in the SOL (*P > *0.05). In the SOL, MYF5 mRNA expression was increased in DOX (*P *<* *0.05), while in the EDL MGF mRNA expression was reduced in DOX (*P *<* *0.05). Chronic DOX administration is associated with reduced fiber size in the SOL and EDL; however, DOX appeared to reduce satellite cell and capillary densities only in the SOL. These findings highlight that therapeutic targets to protect skeletal muscle from DOX may vary across muscles.

## Introduction

Doxorubicin (DOX) is an anthracycline‐class chemotherapeutic agent that is highly effective for treating various cancers (Minotti et al. 2004). However, the clinical utility of DOX is limited due to well‐known off‐target effects (Gilliam et al. 2009; Smuder et al. 2011a,b, 2013; Kavazis et al. 2014), including the induction of skeletal muscle atrophy (Bonifati et al. 2000; Braun et al. 2014) and dysfunction (Hydock et al. 2011; Hayward et al. 2013; Bredahl et al. 2016; Gilliam et al. 2016) that may continue to occur even after the cessation of treatment (Freedman et al. 2004). Importantly, muscle loss is not only associated with reduced strength (Gilliam and St Clair 2011), but it also contributes to fatigue, a major complication of cancer treatment (van Norren et al. 2009). Further, the loss of muscle mass accelerates physical dysfunction and may contribute to the development of heart failure, which is highly prevalent in cancer survivors treated with anthracyclines (Menna et al. 2012; Peel et al. 2014). While the cellular mechanisms through which DOX may exert these deleterious effects on skeletal muscle remain to be fully understood, previous studies have demonstrated increased formation of reactive oxygen species (Gilliam et al. 2016), as well as increased inflammatory (Gilliam et al. 2009, 2011) and proteolytic markers (Smuder et al. 2011a; Kavazis et al. 2014) in skeletal muscle within days following the administration of a large, single bolus of DOX. When administering smaller doses of DOX over biweekly intervals, to mimic the clinical utility of DOX, we have recently shown reduced muscle fiber size to coincide with impaired skeletal muscle mammalian target of rapamycin (mTOR) signaling (Dickinson et al. 2017). Indeed, the mTOR pathway is implicated in a variety of cellular processes related to muscle size including protein translation, cell proliferation, and autophagy (Saxton and Sabatini 2017). Thus, a more precise characterization of the cellular response of skeletal muscle to chronic DOX administration may provide valuable therapeutic targets to reduce toxicities and improve clinical utility.

Since their discovery (Katz 1961; Mauro 1961), satellite cells have been purported to serve an essential role in skeletal muscle growth and regeneration (Kuang and Rudnicki 2008). Indeed, in vitro exposure to DOX has been shown to interfere with myogenic processes associated with satellite cells, including repressed activity of a key myogenic regulatory factor, myogenic differentiation factor 1 (MyoD) (Kurabayashi et al. 1993, 1994), and impaired progression of myoblasts through the myogenic lineage (Necco and Ferraguti 1979; Kurabayashi et al. 1993; Puri et al. 1997). Furthermore, the administration of a single bolus of DOX appears to exacerbate muscle fiber loss when injected during the peak satellite cell activation window following an injury (Nguyen et al. 1998). Satellite cells have also been reported to actively engage in unique crosstalk with neighboring capillaries to facilitate myogenic and angiogenic processes (Christov et al. 2007; Abou‐Khalil et al. 2010). Interestingly, chronic administration of DOX also appears to reduce cardiac muscle capillaries by ~50% (Ammar et al. 2015). While there appears to be a potential role for satellite cells and capillaries as therapeutic targets for attenuating DOX‐induced toxicities in skeletal muscle, the impact of DOX on satellite cell and capillary content, and their relation to muscle fiber size, has not been thoroughly investigated.

Recently, the degree to which satellite cells contribute to the homeostatic maintenance of muscle fiber size in adulthood has garnered review (Murach et al. 2018). Specifically, the role of satellite cells in the maintenance of fiber size may differ between skeletal muscles of various fiber types (Kelly 1978; Gibson and Schultz 1983), functions (Kalhovde et al. 2005) and capillarization (Nederveen et al. 2018). Thus, there not only remains a need to explore the impact of in vivo DOX administration on satellite cells and capillary contents in skeletal muscle, but in particular, there is a need to assess this relationship in skeletal muscles of varying fiber type and function. Therefore, the purpose of this study was to determine the impact of chronic biweekly DOX administration on satellite cell and capillary content in the soleus (SOL) and extensor digitorum longus (EDL) muscles. These muscles were selected as they differ substantially in fiber size (Staron et al. 1999), fiber type (Staron et al. 1999), and capillarization (Murakami et al. 2010), and the function of these muscles appears to be differentially impacted by DOX (Hydock et al. 2011; Hayward et al. 2013). We hypothesized that chronic DOX administration would be associated with reduced satellite cell and capillary content as well as reduced fiber size, in both muscles.

## Materials and Methods

### Study design

Eight‐week‐old ovariectomized female Sprague‐Dawley rats were purchased (Charles River Labs, Wilmington, MA) and acclimated to the laboratory for 1 week as previously described (Dickinson et al. 2017). DOX is considered to be one of the most effective treatments for breast cancer, and therefore, ovariectomized female rats were studied to provide a gonadal estrogen‐deficient model to align with the postmenopausal hormonal environment present in many breast cancer patients in which the median age of diagnoses is 61 years of age (NCI SEER statistics). Rats included in the current study were randomized to receive either Doxorubicin (DOX) or a saline vehicle (VEH). Rats were pair‐housed with an animal in the same group and allowed access to food and water ad libitum. Animals were maintained on a 12‐h light‐12‐h dark cycle and were not provided with any additional means for physical activity (i.e., running wheel). All methods used in this investigation were approved by the Midwestern University Institutional Animal Care and Use Committee.

DOX (doxorubicin hydrochloride >99%; LC Laboratories, Woburn, MA) dissolved in 0.9% normal saline or vehicle (0.9% normal saline) was injected into the rats (I.P.) on three occasions. Injections were separated by two‐week intervals. The dosing strategy was designed to closely mimic strategies used in the clinical treatment of human cancer patients (van Dalen et al. 2016) and has been previously detailed (Dickinson et al. 2017). Briefly, during each injection, DOX was administered in doses of 4 mg∙kg^−1^; totaling 12 mg∙kg^−1^ received by each animal by the end the experimental protocol. Five days following the final injection, rats were anesthetized with isoflurane then decapitated. Upon euthanasia, the SOL and EDL muscles were removed from each leg. The muscles from one leg were immediately frozen in liquid nitrogen for RT‐qPCR analysis while the muscles from the opposite leg were carefully embedded on a foil‐wrapped cork in Tissue Tek optimal cutting temperature medium (OCT; Thermo Fisher Scientific, Rockford, IL) and frozen in liquid nitrogen‐cooled isopentane for immunohistochemical analysis. All samples were stored at −80°C for subsequent analyses. Body weight was assessed at the time of each injection and at time of euthanasia.

### Immunohistochemistry

Seven‐micron‐thick sections were cut in a cryostat (HM525X; ThermoFisher, Waltham, MA), and sections were allowed to air dry for 1 h. Determination of cross‐sectional area (CSA) and fiber type was performed as previously described for these animals, in which fiber type‐specific CSA in the SOL has been previously reported (Dickinson et al. 2017). Briefly, unfixed slides were incubated for 90 min at room temperature with antibodies directed against laminin (no. L9393; Sigma Aldrich, St. Louis, MO), myosin heavy chain (MHC) I [BA.D5; supernatant, Developmental Studies Hybridoma Bank (DSHB, Iowa City, IA)], MHC IIa (SC.71; supernatant, DSHB), and MHC IIb (BF.F3; supernatant, DSHB). Following a series of washes in phosphate‐buffered saline (PBS), slides were incubated for 60 min at room temperature in goat anti‐rabbit AF488 (#A11034; Invitrogen), goat anti‐mouse IgG2b AF488 (#A21141; Invitrogen), goat anti‐mouse IgG1 AF546 (#A21123; Invitrogen), and goat anti‐mouse IgM AF350 (#A31552; Invitrogen) antibodies. Finally, slides were washed in PBS, and mounted with fluorescent mounting media (Fluormount G, #0100‐01; Southern Biotech, Birmingham, AL).

For Pax7/laminin/DAPI staining (visualization of myonuclei and satellite cells), immunohistochemical procedures were adapted from Finnerty et al. (2017). Briefly, slides were fixed in 4% paraformaldehyde (PFA) followed by epitope retrieval using sodium citrate (10 mmol L^−1^, pH 6.5) at 92°C for 20 min. Endogenous peroxidases were blocked with 3% hydrogen peroxide in PBS, and then slides were incubated overnight at 4°C in anti‐laminin (no. L9393; Sigma Aldrich, St. Louis, MO) and anti‐Pax7 antibody (DSHB). The next day, slides were incubated for 70 min at room temperature in goat anti‐rabbit AF488 (#A11034; Invitrogen, laminin) and goat anti‐mouse biotinylated secondary antibody (no. 115‐065‐205; Jackson ImmunoResearch, West Grove, PA), and then reacted with streptavidin‐horseradish peroxidase (HRP) and AF555 tyramide included with the tyramide signal amplification (TSA) kit (#B40933; Invitrogen). TSA‐AF555 was used to visualize Pax7 antibody binding. Slides were co‐stained with 4’,6‐diamidino‐2‐phenylindole (DAPI, #D35471; Invitrogen) before being mounted with Vectashield fluorescent mounting media (Vector Laboratories, Burlingame, CA).

To examine whether DOX administration impacted satellite cell proliferation, we performed Pax7/Ki67 staining. Slides were fixed in 4% PFA for 10 min and then subjected to antigen retrieval in 10 mmol L^−1^ sodium citrate. Slides were then blocked in 2.5% normal horse serum and incubated in mouse anti‐Pax7 and rabbit anti‐Ki67 (no. CRM325B, Biocare Medical, Concord, CA) overnight. The following morning, slides were incubated in AF647 conjugated‐wheat germ agglutinin (WGA, #W32466; Invitrogen), goat anti‐rabbit AF555 (#A21424; Invitrogen) and goat anti‐mouse biotin secondary antibody (no. 115‐065‐205; Jackson ImmunoResearch) for 1 h, and reacted with streptavidin–horseradish peroxidase and AF488 tyramide included with the TSA kit (#T20932, Invitrogen). Slides were co‐stained with DAPI prior to being mounted with Vectashield fluorescent mounting media (Vector Laboratories, Burlingame, CA).

For visualization of capillaries, slides were fixed for 10 min in ice‐cold acetone (−20°C) and then blocked for 1 h in 2.5% normal horse serum (Vector Laboratories). Slides were incubated overnight at 4°C in antibody against CD31/PECAM (#550300, BD Biosciences, San Jose, CA). The next day, slides were incubated for 1 h at room temperature in goat anti‐mouse AF555 (#A21127, Invitrogen) and AF488 conjugated‐WGA (#W11261, Invitrogen).

### Image acquisition and analysis

Images were captured at 100× total magnification at room temperature with a Zeiss upright microscope equipped with an automated stage for image acquisition and analysis of the entire muscle cross section (AxioImager M1; Zeiss, Oberkochen, Germany). The average number of fibers or average muscle area for each analysis is presented with the corresponding results (see Results). Muscle fiber CSA was analyzed from images of laminin staining using a semiautomated threshold analysis (ImageJ, NIH, Bethesda, MD) as previously detailed (Dickinson et al. 2017). Image analysis for satellite cells, Pax7/Ki67+, myonuclei, and capillaries was performed using the AxioVision Rel software (v. 4.9) by a single assessor blinded to group. Satellite cell abundance was assessed as previously described (Finnerty et al. 2017). Briefly, Pax7^+^/DAPI^+^ loci residing within the laminin border were counted as satellite cells. A nucleus was identified as a myonucleus if it met one of the following criteria: (1) it was clearly located within the laminin boundary; (2) it was on the laminin boundary facing inside the fiber; or (3) 50% of the area fell inside the laminin boundary. In accordance with previous reliability analyses (Liu et al. 2013), a minimum of 100 nuclei were counted per muscle cross section. Cellular structures positive for CD31^+^ were counted as capillaries. Our hypothesis was that DOX administration would reduce muscle fiber size, therefore, satellite cells, myonuclei, and capillary counts were normalized to muscle area (mm^2^), while Pax7/Ki67+ cells were normalized to total satellite cell number. However, we also provide these immunohistochemical data normalized to fiber number. To that end, our approach was to compare the effects of DOX administration between distinct muscles, and thus our immunohistochemical assays were not suited to distinguish outcomes specific to muscle fiber types within each muscle. Thus, data normalized to fiber number are independent of fiber type.

### RNA extraction and semiquantitative real‐time PCR

RNA isolation, cDNA synthesis, and real‐time quantitative PCR were performed as our laboratory has previously described (Dickinson et al. 2017). Briefly, frozen muscle tissue was weighed (mean ± SD, 25.84 ± 6.5 mg) at 4°C and homogenized with a hand‐held homogenizing dispenser (Bio‐Gen PRO200, Pro Scientific, Oxford, CT) in 1 mL of Tri reagent (Molecular Research Center, Cincinnati, OH). RNA concentration was determined using a Take3 plate (Biotek, Winooski, VT) and Biotek H1 Synergy. RNA (5 *μ*g) was DNase‐treated using a commercially available kit (DNA‐free, Ambion, Austin, TX). A total of 1 *μ*g of RNA was reverse transcribed into cDNA according to the directions provided by the manufacturer (SensiFAST, Bioline, Taunton, MA). Real‐time qPCR was carried out with a CFX Connect Real‐Time PCR Detection System (BioRad). cDNA was analyzed with SYBR green fluorescence (iTaq Universal SYBR green supermix; BioRad). Primer sequences were designed using the National Center for Biotechnology Information database (Ye et al. 2012) to be mRNA‐specific and compatible with SYBR green chemistry. Primer pairs (Table 1) were purchased from Invitrogen (Carlsbad, CA) and carefully optimized as previously described (D'Lugos et al. 2018). mRNA expression analyses were only targeted to genes associated with myogenesis. *β*_2_‐Microglobulin was utilized as a normalization/housekeeping gene. Relative fold changes from VEH were determined from the Cq values using the 2^−∆∆Ct^ method (Livak and Schmittgen 2001).

**Table 1:** Primer sequences used for real‐time PCR

mRNA	Accession No.	Primer sequence (5’–3’)		Product size (bp)
*Id2*	NM_013060.3	Sense	GGACAGAACCAAACGTCCAG	94
		Antisense	TAAGCTCAGAAGGGAATTCAGAC	
*MGF*	NM_001082478.1	Sense	TGACATGCCCAAGACTCAGAAGT	70
		Antisense	CCTTCTCCTTTGCAGCTTCCT	
*MYF5*	NM_001106783.1	Sense	TGTCTGGTCCCGAAAGAACA	103
		Antisense	CAAGCAATCCAAGCTGGACA	
*MYF6*	NM_013172.2	Sense	CCCTTACAGCTACAAACCCAAG	130
		Antisense	TGCTCCTCCTTCCTTAGCAG	
*MyoD*	NM_176079.1	Sense	GGAGACATCCTCAAGCGATGC	104
		Antisense	GCACCTGGTAAATCGGATTG	
*Myogenin*	NM_017115.2	Sense	CAGTGAATGCAACTCCCACA	85
		Antisense	CAAATGATCTCCTGGGTTGG	
*Myostatin*	NM_019151.1	Sense	GGCAGAGTATTGATGTGAAGAC	119
		Antisense	TGGGAAGGTTACAGCAAGATC	
*Mdm2*	NM_001108099.1	Sense	CCGAGCGAAATGGTCTCTCA	93
		Antisense	CTGCAGACCGCTGCTACTC	
*Npm1*	NM_012992.4	Sense	TCAAGTGCGCGCCTCC	97
		Antisense	CAGCCTTTAGTTCACAACCGAA	
*Rb1*	NM_017045.1	Sense	TGTATGGCATCTGCAAGGTGA	100
		Antisense	CGTTTAAAGGTCTCCTGGGC	

Id2, Inhibitor of DNA Binding 2; MGF, Mechano growth factor; MYF5, Myogenic factor 5; MYF6, Myogenic factor 6; MyoD, Myogenic differentiation factor 1; Mdm2, mouse double minute 2 homolog; Npm1, Nucleophosmin 1; Rb1, Retinablastoma 1.

### Statistical analyses

All data were tested for normality through skewness and kurtosis analyses and visual inspection of the normality plots using SPSS v.24 (IBM). Body mass was analyzed using a two‐way (group × time) ANOVA with repeated measures on the time factor. Satellite cell, myonuclei, capillary, and mRNA expression data within a muscle were analyzed using an independent samples *t*‐test. To account for multiple measures obtained from the same animal, all individual fiber CSA data from an individual animal were nested and data were analyzed using a multi‐level mixed model (nested ANOVA) (Dickinson et al. 2017). This statistical model allows for the inclusion of the multiple measures (i.e., all fibers analyzed), but avoids treating each fiber as an independent observation and takes into account variability for measures obtained both within and between animals. Comparison of the muscle area (mm^2^) examined for immunohistochemical analyses data were compared within each muscle using a two‐sided student's *t*‐test. Statistical analyses were conducted using SPSS v.24 (IBM) and SigmaStat version 12.0 (Systat Software). All critical *P* values were two‐sided and significance was set a priori at *P *≤* *0.05. Data are presented as mean ± standard error (SE) where individual data are not shown.

## Results

### Experimental groups

A significant interaction was observed for body mass (*P *<* *0.001). Body mass was not statistically different between DOX (266 ± 3 g) and VEH (273 ± 3 g) animals at the time of first injection (*P *=* *0.396). However, body mass was greater in VEH versus DOX animals at the time of second injection (289 ± 3 vs. 270 ± 3 g; *P *=* *0.036), time of third injection (303 ± 3 vs. 269 ± 3 g; *P *<* *0.001), and time of sacrifice (304 ± 2 vs. 262 ± 3 g; *P *<* *0.001). Similar to previously reported data (Dickinson et al. 2017), the body mass of VEH animals increased (*P *<* *0.001) until the time of euthanasia, whereas animals receiving DOX exhibited no change in body mass (*P *=* *0.675 for time of first injection to the time of euthanasia).

### Muscle fiber CSA

**Figure 1:**
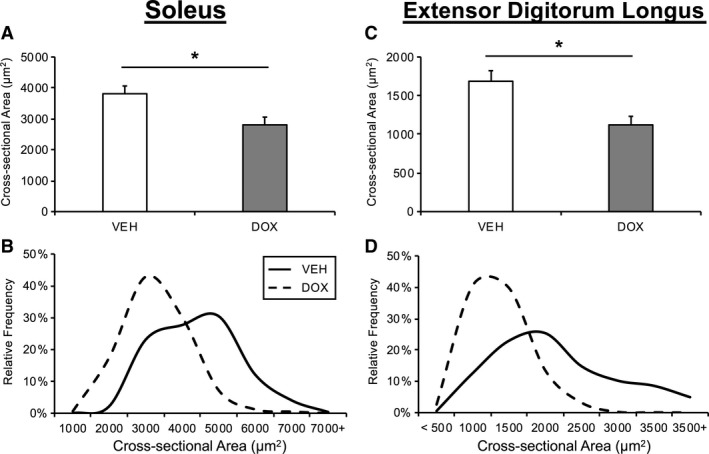
The effect of chronic doxorubicin administration on muscle fiber cross‐sectional area (CSA). Mean fiber CSA of soleus (A) and extensor digitorum longus (C) presented as mean ± SE. Histogram distribution of fiber CSA in soleus (B) and extensor digitorum longus (D). VEH, vehicle group; DOX, doxorubicin group. Soleus (VEH,* n* = 4; DOX,* n* = 4); extensor digitorum longus (VEH,* n* = 4; DOX,* n* = 6). *P <* *0.05 VEH versus DOX.

To assess the impact of DOX administration on muscle fiber size, we examined SOL and EDL fiber CSA. Table 2 displays descriptive data for fiber type profile and CSA for each fiber type among the four groups. Given 1) the influence of DOX on CSA was similar among fiber types in each muscle, and 2) the large discrepancy in fiber type profile between the SOL and EDL, the fiber CSA was pooled across fiber types in each muscle for analyses. Similar to the fiber type‐specific data previously reported (Dickinson et al. 2017), DOX administration was associated with smaller pooled‐fiber CSA in the SOL (26.1 ± 6.2% difference; Fig. 1A; *P *=* *0.022). In addition, fiber CSA in the EDL was smaller in DOX animals (33.2 ± 1.6% difference; Fig. 1C; *P *=* *0.009). The distribution of fiber CSA in each muscle displayed a clear leftward shift, indicating a greater relative abundance of smaller fibers in both the SOL and EDL in DOX animals (Fig. 1B and D). A minimum of 150 SOL fibers and 300 EDL fibers were analyzed per animal.

**Table 2:** Descriptive data for soleus and extensor digitorum longus muscle myosin heavy chain (MHC) profile and cross‐sectional area (CSA)

	MHC I	MHC IIa	MHC IIx	MHC IIb
Soleus				
Percent (%)				
VEH	97.3 ± 1.7	2.7 ± 1.7	–	–
DOX	95.2 ± 2.5	4.8 ± 2.5	–	–
CSA (*μ*m^2^)				
VEH	3539 ± 224	2165 ± 189	–	–
DOX	2873 ± 228	1536 ± 76	–	–
Extensor digitorum longus				
Percent (%)				
VEH	2.7 ± 0.6	6.9 ± 3.7	29.5 ± 10.0	61.0 ± 14.4
DOX	9.2 ± 3.4	28.9 ± 6.1	29.3 ± 2.4	32.6 ± 8.6
CSA (*μ*m^2^)				
VEH	1496 ± 43	1177 ± 400	1419 ± 351	2437 ± 67
DOX	843 ± 102	802 ± 103	1248 ± 100	1452 ± 99

No statistical differences were detected for % fiber type between groups in either muscle (*P* > 0.05). Statistical analyses of CSA are presented in Figure 1. Data are mean ± SE. VEH, vehicle group; DOX, doxorubicin group.

### Satellite cell and myonuclei content

**Figure 2:**
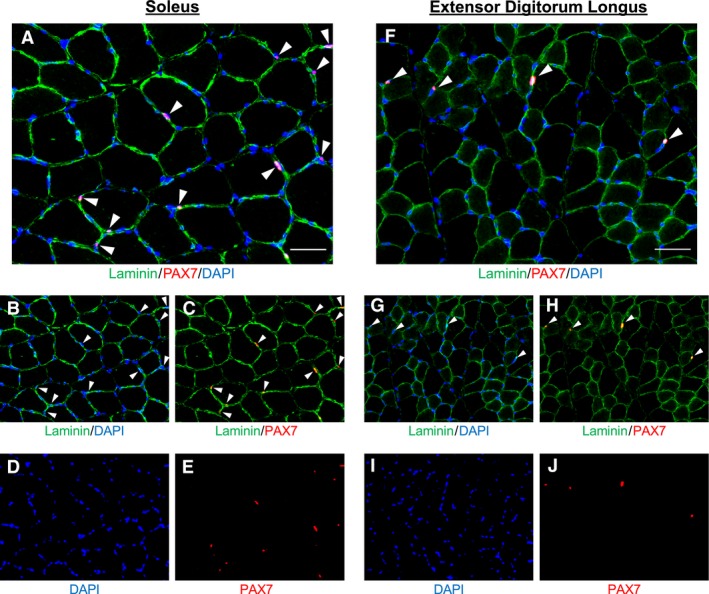
Representative immunohistochemical image of satellite cell detection in the soleus and extensor digitorum longus. Merged images (A,F) demonstrating laminin (green), Pax7 (red), and 4′,6‐diamidino‐2‐phenylindole (DAPI, blue) with satellite cells denoted by white arrowheads. Images captures at 200×, scale bar represents 50 *μ*m. Merged images (B, G) demonstrating satellite cell (red) location within laminin (green), co‐stained with DAPI (blue; C, H) and denoted by white arrowheads. Single‐channel image (D, I) demonstrating DAPI (blue). Single‐channel image (E, J) demonstrating Pax7 (red).

**Figure 3:**
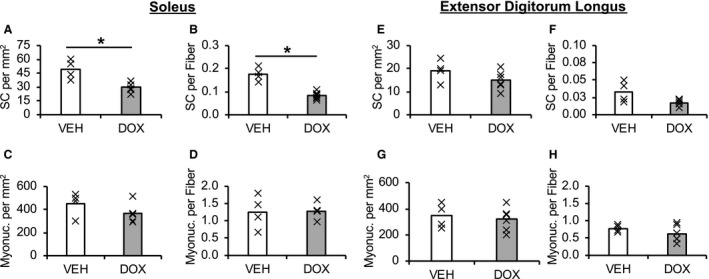
The effect of chronic doxorubicin administration on satellite cell and myonuclei densities. Mean satellite cell density of soleus (A‐B) and extensor digitorum longus (E‐F) presented as mean (bar) with individual animals represented by an “X”. Mean myonuclei density of soleus (C‐D) and extensor digitorum longus (G‐H). SC, satellite cell; VEH, vehicle group; DOX, doxorubicin group. Soleus (satellite cell: VEH,* n* = 4; DOX,* n* = 6; myonuclei: VEH,* n* = 4; DOX,* n* = 5); extensor digitorum longus (satellite cell: VEH,* n* = 4; DOX,* n* = 6; myonuclei: VEH,* n* = 4; DOX,* n* = 6). *P <* *0.05 VEH versus DOX.

**Figure 4:**
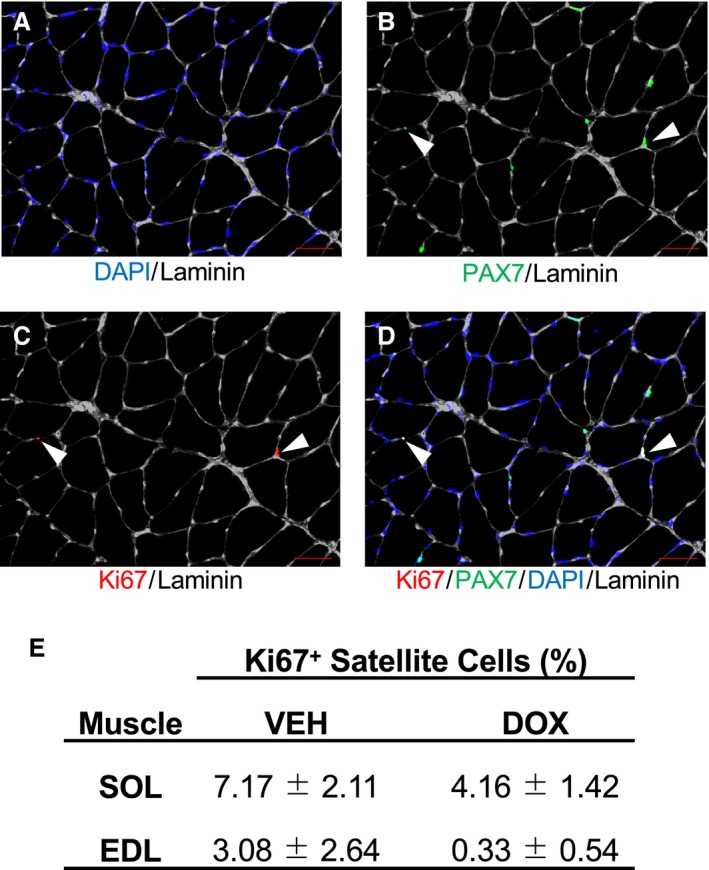
The effect of chronic doxorubicin administration on proliferating satellite cells. Representative immunohistochemical images of proliferating satellite cells (A‐D). (A) DAPI (blue) and laminin (white); (B) PAX7 (green) and laminin (white); (C), Ki67 (red) and laminin (white); (D) merged image denoting Ki67 (red), PAX7 (green), DAPI (blue), and laminin (white). Images capture at 200×, scale bar represents 50 *μ*m. Percentage of proliferating satellite cells (Ki67+) in the soleus and extensor digitorum longus (E) are presented as mean ± SE. VEH, vehicle group; DOX, doxorubicin group; SOL, soleus, EDL, extensor digitorum longus. Soleus (VEH,* n* = 4; DOX,* n* = 7); extensor digitorum longus (VEH,* n* = 4; DOX,* n* = 6).

Representative immunohistochemical images of satellite cell and myonuclei analyses are shown in Figure 2. Pax7‐positive satellite cell content in the SOL was 38 ± 4% lower in DOX versus VEH animals (Pax7+/mm^2^: Fig. 3A, *P *=* *0.025; Pax7+/fiber: Fig. 3B, *P *<* *0.001). Notably, inspection of the individual data demonstrate that the satellite cell content in the SOL of all animals that were administered DOX was lower than every animal in VEH (Fig. 3A and B). Conversely, although numerically reduced, satellite cell content was not statistically impacted by DOX administration in the EDL (Pax7+/mm^2^: Fig. 3E, *P *=* *0.180; Pax7+/fiber: Fig. 3F, *P *=* *0.123). The percentage of Ki67+ satellite cells (Fig. 4) was not impacted by DOX in the SOL (Fig. 4E; *P *=* *0.250). Similarly, the percentage of Ki67+ satellite cells was also not statistically impacted by DOX in the EDL (Fig. 4E; *P *=* *0.127); however, no Ki67+ satellite cells were identified in 4/6 animals in the SED‐DOX group. Myonuclei content was not affected by DOX administration in the SOL (myonuclei/mm^2^: Fig. 3C, *P *=* *0.292; myonuclei/fiber: Fig. 3D, *P *=* *0.917) or EDL (myonuclei/mm^2^: Fig. 3G, *P *=* *0.660; myonuclei/fiber: Fig. 3H, *P *=* *0.966). No difference was detected for the muscle area (mm^2^) analyzed for satellite cell data and myonuclei content for each muscle between groups (*P *>* *0.05; SOL: VEH =3.43 ± 0.35, DOX = 5.03 ± 0.98; EDL: VEH = 5.81 ±0.74, DOX = 5.21 ± 0.53 mm^2^).

### Capillary content

**Figure 5:**
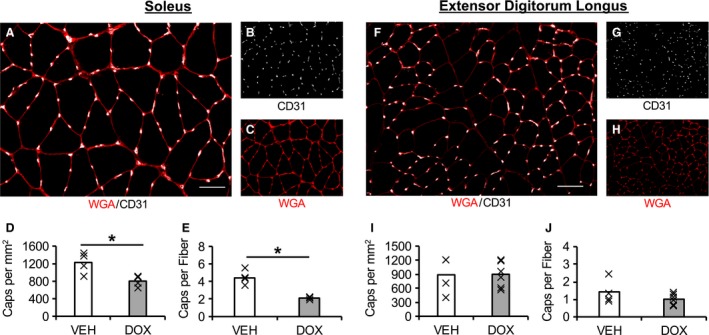
The effect of chronic doxorubicin administration on capillary density. Merged images (A, F) demonstrating CD31 (white) and wheat germ agglutinin (WGA, red). Images captures at 200×, scale bar represents 50 *μ*m. Single‐channel images (B, G) demonstrating CD31 (white). Single‐channel images (C, H) demonstrating WGA (red). Mean capillary density of soleus (D‐E) and extensor digitorum longus (I‐J) presented as mean (bar) with individual animals represented by an “X”. VEH, vehicle group; DOX, doxorubicin group. Soleus (VEH,* n* = 4; DOX,* n* = 4); extensor digitorum longus (VEH,* n* = 4; DOX,* n* = 6). *P <* *0.05 VEH versus DOX.

Capillary content in the SOL was 35 ± 5% lower in DOX versus VEH animals (CD31/mm^2^: Fig. 5D, *P *=* *0.021; CD31/fiber: Fig. 5E, *P *=* *0.002). Notably, inspection of the individual data demonstrates that the SOL capillary content of all animals that were administered DOX was lower than every VEH animal (Fig. 5D, 5E). In contrast, capillary content of the EDL was not impacted by DOX administration (CD31/mm^2^: Fig. 5I, *P *=* *0.959; CD31/fiber: Fig. 5J, *P *=* *0.234). No difference was detected for the muscle area (mm^2^) analyzed for capillary content for each muscle across treatments (*P *>* *0.05; SOL: VEH = 1.23 ± 0.47, DOX = 5.50 ± 1.92; EDL: VEH =3.64 ± 1.05, DOX = 2.89 ± 0.54 mm^2^).

### Myogenic gene expression

The mRNA expression of MYF5 was higher in animals administered DOX only in the SOL (*P *=* *0.026). MGF mRNA expression was lower in animals administered DOX only in the EDL (*P *=* *0.048). No differences were detected for the mRNA expression of several other genes associated with myogenesis in the SOL and EDL (Table 3).

**Table 3:** Soleus and extensor digitorum longus muscle mRNA analyses for markers of myogenic activity

mRNA	Soleus		Extensor digitorum longus	
	VEH	DOX	VEH	DOX
*Id2*	1.24 ± 0.33	1.30 ± 0.24	1.03 ± 0.14	0.92 ± 0.16
*Mdm2*	1.05 ± 0.42	0.76 ± 0.21	1.08 ± 0.25	0.79 ± 0.24
*MGF*	1.25 ± 0.30	1.18 ± 0.13	1.01 ± 0.07	0.80 ± 0.01*
*MYF5*	1.07 ± 0.16	1.59 ± 0.13*	1.02 ± 0.11	0.80 ± 0.01
*MYF6*	1.08 ± 0.16	0.89 ± 0.09	1.14 ± 0.34	0.54 ± 0.04
*MYOD*	1.25 ± 0.35	1.90 ± 0.23	1.18 ± 0.40	0.57 ± 0.08
*Myogenin*	1.43 ± 0.54	1.25 ± 0.15	1.16 ± 0.39	0.96 ± 0.17
*Myostatin*	1.38 ± 0.38	1.31 ± 0.25	1.18 ± 0.43	0.90 ± 0.24
*Npm1*	1.31 ± 0.49	0.94 ± 0.11	1.02 ± 0.12	1.16 ± 0.19
*Rb1*	1.23 ± 0.38	0.78 ± 0.11	1.03 ± 0.15	0.93 ± 0.15

Data are mean ± SE. Values represent relative fold change from vehicle group (VEH) as determined from the Cq values using the 2–ΔΔCt method (Livak and Schmittgen 2001). *P < 0.05 VEH versus doxorubicin group (DOX). Id2, Inhibitor of DNA Binding 2; MGF, Mechano growth factor; MYF5, Myogenic factor 5; MYF6, Myogenic factor 6; MyoD, Myogenic differentiation factor 1; Mdm2, mouse double minute 2 homolog; Npm1, Nucleophosmin 1; Rb1, Retinablastoma 1. Soleus: VEH, *n* = 8; DOX, *n* = 8; EDL: VEH, *n* = 4; DOX, *n* = 6.

## Discussion

The purpose of the current study was to determine the impact of chronic DOX administration on satellite cell and capillary densities in different skeletal muscles. Several novel findings resulted from this study. First, chronic DOX administration was associated with lower skeletal muscle satellite cell density in the SOL. The lower satellite cell density in the SOL of DOX animals coincided with lower capillary density and reduced muscle fiber CSA. Conversely, while chronic DOX administration was also associated with reduced CSA in the EDL, satellite cell and capillary densities were less impacted. These preliminary findings suggest that (1) DOX impairs the regulation of muscle fiber CSA across different muscles of varying phenotype, (2) DOX administration may impact satellite cell and capillary densities in a muscle‐specific manner, (3) the role of satellite cells in the regulation of muscle fiber size may differ across muscles.

We have previously reported reduced MHC I and MHC IIa fiber CSA in the SOL of animals that underwent chronic DOX administration (Dickinson et al. 2017). DOX has been shown to impact the function of both the SOL and EDL (Hydock et al. 2011; Hayward et al. 2013), however, to what extent chronic DOX administration impacts fiber size in the EDL has not been investigated. In the current study, chronic DOX administration was associated with a similar relative reduction in fiber CSA in both the SOL and EDL muscles despite the fact that these muscles differ considerably in their fiber type profile (Kelly 1978; Gibson and Schultz 1983), fiber size (Staron et al. 1999), function (Kalhovde et al. 2005), capillarization (Nederveen et al. 2018), and potentially their accumulation of DOX (Hayward et al. 2013). However, while chronic DOX administration reduced muscle fiber size in both the SOL and EDL, in this study satellite cell density was only found to be reduced in the SOL. Satellite cells are understood to contribute to the regulation of muscle fiber size (Keefe et al. 2015); however, the role of satellite cells in maintaining fiber size may differ depending on the muscular environment in which they reside. Indeed, the work of Kelly (1978) propose that fiber size in the SOL is more closely associated with satellite cell fusion whereas fiber size in the EDL is more a product of cytoplasmic expansion. Interestingly, MGF mRNA expression, which has been shown to be regulated by circulating growth hormone (Iida et al. 2004) and positively correlated to lean mass (Pollanen et al. 2010), was reduced by DOX only in the EDL muscle. Therefore, the DOX‐induced reduction in fiber size of the EDL could also reflect impairments in processes of myocellular growth that are less reliant on satellite cells (i.e., protein synthesis). In addition, previous reports indicate satellite cells and myonuclei within the EDL are less mitotically and transcriptionally active, respectively, compared to those within SOL (Goldberg 1967; Kelly 1978). Thus, it is interesting to speculate that perhaps the greater mitotic activity of satellite cells in the SOL, relative to those in the EDL, could make them more susceptible to DOX. Collectively, our data indicate that the role of satellite cells, as it relates to fiber size, could differ between the SOL and EDL.

A primary role of satellite cells is to fuse to existing fibers and donate their nuclei as a source of new myonuclei (Moss and Leblond 1971). Despite reduced satellite cell density in the SOL of animals receiving DOX, myonuclear density was not affected in either muscle following DOX administration. Previous research has shown that a single bolus injection of DOX increases the occurrence of apoptotic myonuclei in skeletal muscle (Smuder et al. 2011a). Thus, to maintain the myonuclear pool in the presence of DOX‐induced myonuclear apoptosis, satellite cells must activate and proliferate. On the other hand, DOX is also a known inhibitor of cell proliferation (Hanusova et al. 2011) and directly inhibits the activity of topoisomerase II (Tewey et al. 1984), with peak expression levels during G2/M phase of the cell cycle (Larsen et al. 1996) along with that of Ki67. Therefore, to determine the extent to which changes in satellite cell proliferation were potentially impacting the myonuclear pool, we examined Ki67+ satellite cells. Interestingly, we did not observe an impact of chronic DOX administration on satellite cell proliferation in the SOL. Therefore, it appears that the diminishing satellite cell population in the SOL during chronic DOX administration is not a result of impaired proliferation. Indeed, this interpretation is supported by increased mRNA expression of Myf5 in the SOL of animals administered DOX, which is involved in satellite cell proliferation (Yin et al. 2013) and self‐renewal (Sabourin et al. 1999). Collectively, these findings further highlight the intricacies in the regulation of satellite cell and myonuclear abundance, and in particular, these data emphasize the need for future research focused to identify the mechanisms regulating satellite cell dynamics in skeletal muscles with dissimilar traits, and how they are affected by myotoxic drugs such as DOX.

Mounting evidence indicates that satellite cells communicate with capillaries through paracrine interactions to facilitate myogenic and angiogenic processes (Christov et al. 2007; Abou‐Khalil et al. 2010). Indeed, satellite cells and capillaries appear to share a general proximity within skeletal muscle (Christov et al. 2007; Nederveen et al. 2016). Given the vital role of skeletal muscle capillaries in delivering oxygen, substrates and circulating factors, and their proposed relationship with satellite cells, we also examined capillary density in response to DOX. Similar to satellite cell density, we also show that DOX administration resulted in reduced capillary density in the SOL, but not the EDL. Our findings in the SOL are consistent with a previous study (Ammar et al. 2015) demonstrating reduced capillaries in the myocardium of diabetic rats following chronic DOX administration. Interestingly, in this previous study myocardial capillary content was increased when stem cells were administered along with DOX (Ammar et al. 2015). In addition, in vitro models have also demonstrated capillary density (Rahbarghazi et al. 2013) and capillary growth (Rhoads et al. 2009) are greater in the presence of satellite cells. Indeed, it is possible that the preservation of capillary density in the EDL, which was not seen in the SOL or in previous investigation of the myocardium (Ammar et al. 2015), is related to better preservation of satellite cell content per area of muscle. It is also interesting to speculate that the EDL possesses a distinct ability to preserve capillarization; that is the predominantly fast‐twitch EDL may undergo a remodeling toward a more slow‐twitch phenotype, which is associated with greater capillarization. While speculative, the EDL of animals administered DOX exhibited a drop, albeit not statistically significant, in the percentage of MHC IIb fibers along with a numerical increase in the percentage of MHC I and IIa fibers compared to VEH (Table 3). While the mechanisms involved in capillary‐satellite cell crosstalk are still not fully understood, recent findings purport that satellite cells may recruit endothelial cells through paracrine VEGFA signaling, which in return aid in the maintenance of the satellite cell pool (Verma et al. 2018). Future preclinical studies, such as skeletal muscle satellite cell depletion models, are needed to further discern the role of capillary‐satellite cell crosstalk in the maintenance of muscle fiber size during DOX‐treatment. Nonetheless, the findings from the current study provide further support that the deleterious effects of DOX may occur in a muscle‐dependent manner.

The current study does include some limitations. We acknowledge the preliminary nature of our findings, given the sample size presented for some immunohistochemical analyses. Not every sample met our high standards for tissue quality. Thus, to allow for greater transparency and strength for interpreting our findings, individual animal data are presented for all key immunohistochemical analyses. In addition, dietary intake was not measured in the current study. While food intake has been shown to be reduced following a large bolus administration of DOX in mice (Gilliam et al. 2009), dietary intake has also been shown to not be reduced in rats treated chronically with DOX (Hajjaji et al. 2012). Nonetheless, future studies are needed to discern the impact of changes in dietary‐intake on chemotherapy‐induced myotoxicity. The current study design euthanatized animals and collected tissues five days following the last injection of DOX, which may have impacted some of our results (i.e., mRNA expression). This approach was based on previously published findings showing DOX accumulates in skeletal muscle and causes dysfunction for up to five days following an i.p. injection (Hayward et al. 2013). To what extent the changes in satellite cell activity and content are due to the acute effects of the final DOX injection or the cumulative effect of chronic DOX administration requires further investigation. Lastly, while our findings provide novel insight into the divergent impact of chronic DOX administration on satellite cell and capillary populations in different skeletal muscles, the current study is not able to identify the direct cellular mechanisms through which DOX exerts its muscle‐specific effect on satellite cells and capillaries. While the proposed mechanisms of action for DOX include intercalation into DNA, inhibiting topoisomerase II, and the generation of reactive oxygen species (Gewirtz 1999), to what extent these or other molecular actions contribute to changes in satellite cell and capillary densities requires further investigation. However, our findings indicate that such experiments are likely to be strengthened through examination of multiple skeletal muscles and/or tissues.

In conclusion, we show for the first time that chronic DOX administration is associated with reduced satellite cell and capillary densities in a muscle‐specific manner. Specifically, while DOX administration was associated with reduced muscle fiber size in both the SOL and EDL, satellite cell and capillary densities were found to be reduced by chronic DOX administration only in the SOL. These findings indicate that varying skeletal muscles may rely on different mechanisms to maintain fiber size and that chronic DOX administration may impact fiber size of various skeletal muscles through different cellular mechanisms. While the current study did not examine functional parameters of these muscles, DOX appears to cause dysfunction in both the SOL and EDL (Hydock et al. 2011; Hayward et al. 2013). Coupled with previous findings in which chronic DOX administration attenuated mTORC1 signaling (Dickinson et al. 2017) and mitochondrial dysfunction (Gouspillou et al. 2015), these findings contribute to a growing body of literature highlighting the myotoxicity associated with DOX. Future studies are necessary to examine skeletal muscle satellite cells and capillaries as therapeutic targets in mitigating the deleterious impact of DOX in skeletal muscle. Such insight will dramatically improve our ability to identify and develop effective therapies aimed at protecting skeletal muscle during DOX administration.

## Conflicts of Interests

None declared.

## References

[phy214052-bib-0001] Abou‐Khalil, R. , R. Mounier , and B. Chazaud . 2010 Regulation of myogenic stem cell behavior by vessel cells: the “menage a trois” of satellite cells, periendothelial cells and endothelial cells. Cell Cycle 9:892–896. 20160472 10.4161/cc.9.5.10851

[phy214052-bib-0002] Ammar, H. I. , G. L. Sequiera , M. B. Nashed , R. I. Ammar , H. M. Gabr , H. E. Elsayed , et al. 2015 Comparison of adipose tissue‐ and bone marrow‐ derived mesenchymal stem cells for alleviating doxorubicin‐induced cardiac dysfunction in diabetic rats. Stem Cell Res. Ther. 6:148. 26296856 10.1186/s13287-015-0142-x PMC4546321

[phy214052-bib-0003] Bonifati, D. M. , C. Ori , C. R. Rossi , S. Caira , M. Fanin , and C. Angelini . 2000 Neuromuscular damage after hyperthermic isolated limb perfusion in patients with melanoma or sarcoma treated with chemotherapeutic agents. Cancer Chemother. Pharmacol. 46:517–522. 11138466 10.1007/s002800000175

[phy214052-bib-0004] Braun, T. P. , M. Szumowski , P. R. Levasseur , A. J. Grossberg , X. Zhu , A. Agarwal , et al. 2014 Muscle atrophy in response to cytotoxic chemotherapy is dependent on intact glucocorticoid signaling in skeletal muscle. PLoS ONE 9:e106489. 25254959 10.1371/journal.pone.0106489 PMC4177815

[phy214052-bib-0005] Bredahl, E. C. , K. B. Pfannenstiel , C. J. Quinn , R. Hayward , and D. S. Hydock . 2016 Effects of exercise on doxorubicin‐induced skeletal muscle dysfunction. Med. Sci. Sports Exerc. 48:1468–1473. 27015384 10.1249/MSS.0000000000000926

[phy214052-bib-0006] Christov, C. , F. Chretien , R. Abou‐Khalil , G. Bassez , G. Vallet , F. J. Authier , et al. 2007 Muscle satellite cells and endothelial cells: close neighbors and privileged partners. Mol. Biol. Cell 18:1397–1409. 17287398 10.1091/mbc.E06-08-0693 PMC1838982

[phy214052-bib-0007] van Dalen, E. C. , H. J. van der Pal , and L. C. Kremer . 2016 Different dosage schedules for reducing cardiotoxicity in people with cancer receiving anthracycline chemotherapy. Cochrane Database Syst. Rev. 3:CD005008. 26938118 10.1002/14651858.CD005008.pub4 PMC6457744

[phy214052-bib-0008] Dickinson, J. M. , A. C. D'Lugos , T. N. Mahmood , J. C. Ormsby , L. Salvo , W. L. Dedmon , et al. 2017 Exercise protects skeletal muscle during chronic doxorubicin administration. Med. Sci. Sports Exerc. 49:2394–2403. 28767526 10.1249/MSS.0000000000001395

[phy214052-bib-0009] D'Lugos, A. C. , S. H. Patel , J. C. Ormsby , D. P. Curtis , C. S. Fry , C. C. Carroll , et al. 2018 Prior acetaminophen consumption impacts the early adaptive cellular response of human skeletal muscle to resistance exercise. J. Appl. Physiol. (1985) 124:1012–1024. 29357482 10.1152/japplphysiol.00922.2017

[phy214052-bib-0010] Finnerty, C. C. , C. F. McKenna , L. A. Cambias , C. R. Brightwell , A. Prasai , Y. Wang , et al. 2017 Inducible satellite cell depletion attenuates skeletal muscle regrowth following a scald‐burn injury. J. Physiol. 595:6687–6701. 28833130 10.1113/JP274841 PMC5663820

[phy214052-bib-0011] Freedman, R. J. , N. Aziz , D. Albanes , T. Hartman , D. Danforth , S. Hill , et al. 2004 Weight and body composition changes during and after adjuvant chemotherapy in women with breast cancer. J. Clin. Endocrinol. Metab. 89:2248–2253. 15126549 10.1210/jc.2003-031874

[phy214052-bib-0012] Gewirtz, D. A. 1999 A critical evaluation of the mechanisms of action proposed for the antitumor effects of the anthracycline antibiotics adriamycin and daunorubicin. Biochem. Pharmacol. 57:727–741. 10075079 10.1016/s0006-2952(98)00307-4

[phy214052-bib-0013] Gibson, M. C. , and E. Schultz . 1983 Age‐related differences in absolute numbers of skeletal muscle satellite cells. Muscle Nerve 6:574–580. 6646160 10.1002/mus.880060807

[phy214052-bib-0014] Gilliam, L. A. , and D. K. St Clair . 2011 Chemotherapy‐induced weakness and fatigue in skeletal muscle: the role of oxidative stress. Antioxid. Redox Signal. 15:2543–2563. 21457105 10.1089/ars.2011.3965 PMC3176345

[phy214052-bib-0015] Gilliam, L. A. , L. F. Ferreira , J. D. Bruton , J. S. Moylan , H. Westerblad , D. K. St Clair , et al. 2009 Doxorubicin acts through tumor necrosis factor receptor subtype 1 to cause dysfunction of murine skeletal muscle. J. Appl. Physiol. (1985) 107:1935–1942. 19779154 10.1152/japplphysiol.00776.2009 PMC2793196

[phy214052-bib-0016] Gilliam, L. A. , J. S. Moylan , L. F. Ferreira , and M. B. Reid . 2011 TNF/TNFR1 signaling mediates doxorubicin‐induced diaphragm weakness. Am. J. Physiol. Lung Cell. Mol. Physiol. 300:L225–L231. 21097524 10.1152/ajplung.00264.2010 PMC3043812

[phy214052-bib-0017] Gilliam, L. A. , D. S. Lark , L. R. Reese , M. J. Torres , T. E. Ryan , C. T. Lin , et al. 2016 Targeted overexpression of mitochondrial catalase protects against cancer chemotherapy‐induced skeletal muscle dysfunction. Am. J. Physiol. Endocrinol. Metab. 311:E293–E301. 27329802 10.1152/ajpendo.00540.2015 PMC5005971

[phy214052-bib-0018] Goldberg, A. L. 1967 Protein synthesis in tonic and phasic skeletal muscles. Nature 216:1219–1220. 6076071 10.1038/2161219a0

[phy214052-bib-0019] Gouspillou, G. , C. Scheede‐Bergdahl , S. Spendiff , M. Vuda , B. Meehan , H. Mlynarski , et al. 2015 Anthracycline‐containing chemotherapy causes long‐term impairment of mitochondrial respiration and increased reactive oxygen species release in skeletal muscle. Sci. Rep. 5:8717. 25732599 10.1038/srep08717 PMC4346812

[phy214052-bib-0020] Hajjaji, N. , C. Couet , P. Besson , and P. Bougnoux . 2012 DHA effect on chemotherapy‐induced body weight loss: an exploratory study in a rodent model of mammary tumors. Nutr. Cancer 64:1000–1007. 23035949 10.1080/01635581.2012.714832

[phy214052-bib-0021] Hanusova, V. , I. Bousova , and L. Skalova . 2011 Possibilities to increase the effectiveness of doxorubicin in cancer cells killing. Drug Metab. Rev. 43:540–557. 21942373 10.3109/03602532.2011.609174

[phy214052-bib-0022] Hayward, R. , D. Hydock , N. Gibson , S. Greufe , E. Bredahl , and T. Parry . 2013 Tissue retention of doxorubicin and its effects on cardiac, smooth, and skeletal muscle function. J. Physiol. Biochem. 69:177–187. 22890792 10.1007/s13105-012-0200-0

[phy214052-bib-0023] Hydock, D. S. , C. Y. Lien , B. T. Jensen , C. M. Schneider , and R. Hayward . 2011 Characterization of the effect of in vivo doxorubicin treatment on skeletal muscle function in the rat. Anticancer Res. 31:2023–2028. 21737618

[phy214052-bib-0024] Iida, K. , E. Itoh , D. S. Kim , J. P. del Rincon , K. T. Coschigano , J. J. Kopchick , et al. 2004 Muscle mechano growth factor is preferentially induced by growth hormone in growth hormone‐deficient lit/lit mice. J. Physiol. 560:341–349. 15308683 10.1113/jphysiol.2004.069500 PMC1665252

[phy214052-bib-0025] Kalhovde, J. M. , R. Jerkovic , I. Sefland , C. Cordonnier , E. Calabria , S. Schiaffino , et al. 2005 “Fast” and “slow” muscle fibres in hindlimb muscles of adult rats regenerate from intrinsically different satellite cells. J. Physiol. 562:847–857. 15564285 10.1113/jphysiol.2004.073684 PMC1665547

[phy214052-bib-0026] Katz, B. 1961 The terminations of the afferent nerve fibre in the muscle spindle of the frog. Philos. Trans. R. Soc. Lond. B Biol. Sci. 243:221–240.

[phy214052-bib-0027] Kavazis, A. N. , A. J. Smuder , and S. K. Powers . 2014 Effects of short‐term endurance exercise training on acute doxorubicin‐induced FoxO transcription in cardiac and skeletal muscle. J. Appl. Physiol. (1985) 117:223–230. 24947024 10.1152/japplphysiol.00210.2014 PMC4347740

[phy214052-bib-0028] Keefe, A. C. , J. A. Lawson , S. D. Flygare , Z. D. Fox , M. P. Colasanto , S. J. Mathew , et al. 2015 Muscle stem cells contribute to myofibres in sedentary adult mice. Nat. Commun. 6:7087. 25971691 10.1038/ncomms8087 PMC4435732

[phy214052-bib-0029] Kelly, A. M. 1978 Satellite cells and myofiber growth in the rat soleus and extensor digitorum longus muscles. Dev. Biol. 65:1–10. 680348 10.1016/0012-1606(78)90174-4

[phy214052-bib-0030] Kuang, S. , and M. A. Rudnicki . 2008 The emerging biology of satellite cells and their therapeutic potential. Trends Mol. Med. 14:82–91. 18218339 10.1016/j.molmed.2007.12.004

[phy214052-bib-0031] Kurabayashi, M. , R. Jeyaseelan , and L. Kedes . 1993 Antineoplastic agent doxorubicin inhibits myogenic differentiation of C2 myoblasts. J. Biol. Chem. 268:5524–5529. 8449915

[phy214052-bib-0032] Kurabayashi, M. , R. Jeyaseelan , and L. Kedes . 1994 Doxorubicin represses the function of the myogenic helix‐loop‐helix transcription factor MyoD. Involvement of Id gene induction. J. Biol. Chem. 269:6031–6039. 8119948

[phy214052-bib-0033] Larsen, A. K. , A. Skladanowski , and K. Bojanowski . 1996 The roles of DNA topoisomerase II during the cell cycle. Prog. Cell cycle Res. 2:229–239. 9552399 10.1007/978-1-4615-5873-6_22

[phy214052-bib-0034] Liu, F. , C. S. Fry , J. Mula , J. R. Jackson , J. D. Lee , C. A. Peterson , et al. 2013 Automated fiber‐type‐specific cross‐sectional area assessment and myonuclei counting in skeletal muscle. J. Appl. Physiol. (1985) 115:1714–1724. 24092696 10.1152/japplphysiol.00848.2013 PMC3882739

[phy214052-bib-0035] Livak, K. J. , and T. D. Schmittgen . 2001 Analysis of relative gene expression data using real‐time quantitative PCR and the 2(‐Delta Delta C(T)) Method. Methods 25:402–408. 11846609 10.1006/meth.2001.1262

[phy214052-bib-0036] Mauro, A. 1961 Satellite cell of skeletal muscle fibers. J. Biophys. Biochem. Cytol. 9:493–495. 13768451 10.1083/jcb.9.2.493 PMC2225012

[phy214052-bib-0037] Menna, P. , O. G. Paz , M. Chello , E. Covino , E. Salvatorelli , and G. Minotti . 2012 Anthracycline cardiotoxicity. Expert Opin. Drug Saf. 11(Suppl 1):S21–S36. 21635149 10.1517/14740338.2011.589834

[phy214052-bib-0038] Minotti, G. , P. Menna , E. Salvatorelli , G. Cairo , and L. Gianni . 2004 Anthracyclines: molecular advances and pharmacologic developments in antitumor activity and cardiotoxicity. Pharmacol. Rev. 56:185–229. 15169927 10.1124/pr.56.2.6

[phy214052-bib-0039] Moss, F. P. , and C. P. Leblond . 1971 Satellite cells as the source of nuclei in muscles of growing rats. Anat. Rec. 170:421–435. 5118594 10.1002/ar.1091700405

[phy214052-bib-0040] Murach, K. A. , C. S. Fry , T. J. Kirby , J. R. Jackson , J. D. Lee , S. H. White , et al. 2018 Starring or supporting role? Satellite cells and skeletal muscle fiber size regulation. Physiology 33:26–38. 29212890 10.1152/physiol.00019.2017 PMC5866409

[phy214052-bib-0041] Murakami, S. , H. Fujino , I. Takeda , R. Momota , K. Kumagishi , and A. Ohtsuka . 2010 Comparison of capillary architecture between slow and fast muscles in rats using a confocal laser scanning microscope. Acta Med. Okayama 64:11–18. 20200579 10.18926/AMO/32859

[phy214052-bib-0042] Necco, A. , and M. Ferraguti . 1979 Influence of doxorubicin on myogenic cell fusion. Exp. Mol. Pathol. 31:353–360. 467624 10.1016/0014-4800(79)90036-4

[phy214052-bib-0043] Nederveen, J. P. , S. Joanisse , T. Snijders , V. Ivankovic , S. K. Baker , S. M. Phillips , et al. 2016 Skeletal muscle satellite cells are located at a closer proximity to capillaries in healthy young compared with older men. J. Cachexia Sarcopenia Muscle 7:547–554. 27239425 10.1002/jcsm.12105 PMC4864218

[phy214052-bib-0044] Nederveen, J. P. , S. Joanisse , T. Snijders , A. C. Q. Thomas , D. Kumbhare , and G. Parise . 2018 The influence of capillarization on satellite cell pool expansion and activation following exercise‐induced muscle damage in healthy young men. J. Physiol. 596:1063–1078. 29315567 10.1113/JP275155 PMC5851891

[phy214052-bib-0045] Nguyen, L. T. , L. K. McLoon , and J. D. Wirtschafter . 1998 Doxorubicin chemomyectomy is enhanced when performed two days following bupivacaine injections: the effect coincides with the peak of muscle satellite cell division. Invest. Ophthalmol. Vis. Sci. 39:203–206. 9430564

[phy214052-bib-0046] van Norren, K. , A. van Helvoort , J. M. Argiles , S. van Tuijl , K. Arts , M. Gorselink , et al. 2009 Direct effects of doxorubicin on skeletal muscle contribute to fatigue. Br. J. Cancer 100:311–314. 19165199 10.1038/sj.bjc.6604858 PMC2634729

[phy214052-bib-0047] Peel, A. B. , S. M. Thomas , K. Dittus , L. W. Jones , and S. G. Lakoski . 2014 Cardiorespiratory fitness in breast cancer patients: a call for normative values. J. Am. Heart Assoc. 3:e000432. 24419734 10.1161/JAHA.113.000432 PMC3959685

[phy214052-bib-0048] Pollanen, E. , P. H. Ronkainen , M. Horttanainen , T. Takala , J. Puolakka , H. Suominen , et al. 2010 Effects of combined hormone replacement therapy or its effective agents on the IGF‐1 pathway in skeletal muscle. Growth Horm. IGF Res. 20:372–379. 20724185 10.1016/j.ghir.2010.07.003

[phy214052-bib-0049] Puri, P. L. , S. Medaglia , L. Cimino , C. Maselli , A. Germani , E. De Marzio , et al. 1997 Uncoupling of p21 induction and MyoD activation results in the failure of irreversible cell cycle arrest in doxorubicin‐treated myocytes. J. Cell. Biochem. 66:27–36. 9215525 10.1002/(sici)1097-4644(19970701)66:1<27::aid-jcb4>3.0.co;2-#

[phy214052-bib-0050] Rahbarghazi, R. , S. M. Nassiri , P. Khazraiinia , A. M. Kajbafzadeh , S. H. Ahmadi , E. Mohammadi , et al. 2013 Juxtacrine and paracrine interactions of rat marrow‐derived mesenchymal stem cells, muscle‐derived satellite cells, and neonatal cardiomyocytes with endothelial cells in angiogenesis dynamics. Stem Cells Dev. 22:855–865. 23072248 10.1089/scd.2012.0377 PMC3585743

[phy214052-bib-0051] Rhoads, R. P. , R. M. Johnson , C. R. Rathbone , X. Liu , C. Temm‐Grove , S. M. Sheehan , et al. 2009 Satellite cell‐mediated angiogenesis in vitro coincides with a functional hypoxia‐inducible factor pathway. Am. J. Physiol. Cell Physiol. 296:C1321–C1328. 19386789 10.1152/ajpcell.00391.2008 PMC2692418

[phy214052-bib-0052] Sabourin, L. A. , A. Girgis‐Gabardo , P. Seale , A. Asakura , and M. A. Rudnicki . 1999 Reduced differentiation potential of rrimaryMyoD−/− myogenic cells derived from adult skeletal muscle. J. Cell Biol. 144:631–643. 10037786 10.1083/jcb.144.4.631 PMC2132931

[phy214052-bib-0053] Saxton, R. A. , and D. M. Sabatini . 2017 mTOR signaling in growth, metabolism, and disease. Cell 168:960–976. 28283069 10.1016/j.cell.2017.02.004 PMC5394987

[phy214052-bib-0054] Smuder, A. J. , A. N. Kavazis , K. Min , and S. K. Powers . 2011a Exercise protects against doxorubicin‐induced markers of autophagy signaling in skeletal muscle. J. Appl. Physiol. (1985) 111:1190–1198. 21778418 10.1152/japplphysiol.00429.2011

[phy214052-bib-0055] Smuder, A. J. , A. N. Kavazis , K. Min , and S. K. Powers . 2011b Exercise protects against doxorubicin‐induced oxidative stress and proteolysis in skeletal muscle. J. Appl. Physiol. (1985) 110:935–942. 21310889 10.1152/japplphysiol.00677.2010 PMC3075128

[phy214052-bib-0056] Smuder, A. J. , A. N. Kavazis , K. Min , and S. K. Powers . 2013 Doxorubicin‐induced markers of myocardial autophagic signaling in sedentary and exercise trained animals. J. Appl. Physiol. (1985) 115:176–185. 23703114 10.1152/japplphysiol.00924.2012

[phy214052-bib-0057] Staron, R. S. , W. J. Kraemer , R. S. Hikida , A. C. Fry , J. D. Murray , and G. E. Campos . 1999 Fiber type composition of four hindlimb muscles of adult Fisher 344 rats. Histochem. Cell Biol. 111:117–123. 10090572 10.1007/s004180050341

[phy214052-bib-0058] Tewey, K. M. , T. C. Rowe , L. Yang , B. D. Halligan , and L. F. Liu . 1984 Adriamycin‐induced DNA damage mediated by mammalian DNA topoisomerase II. Science 226:466–468. 6093249 10.1126/science.6093249

[phy214052-bib-0059] Verma, M. , Y. Asakura , B. S. R. Murakonda , T. Pengo , C. Latroche , B. Chazaud , et al. 2018 Muscle satellite cell cross‐talk with a vascular niche maintains quiescence via VEGF and notch signaling. Cell Stem Cell 23:530–543. 30290177 10.1016/j.stem.2018.09.007 PMC6178221

[phy214052-bib-0060] Ye, J. , G. Coulouris , I. Zaretskaya , I. Cutcutache , S. Rozen , and T. L. Madden . 2012 Primer‐BLAST: a tool to design target‐specific primers for polymerase chain reaction. BMC Bioinformatics 13:134. 22708584 10.1186/1471-2105-13-134 PMC3412702

[phy214052-bib-0061] Yin, H. , F. Price , and M. A. Rudnicki . 2013 Satellite cells and the muscle stem cell niche. Physiol. Rev. 93:23–67. 23303905 10.1152/physrev.00043.2011 PMC4073943

